# Supplementary material for: Plakophilin-2 Haploinsufficiency Causes Calcium Handling Deficits and Modulates the Cardiac Response Towards Stress
Source: Int J Mol Sci. 2019 Aug 21;20(17):4076. doi: 10.3390/ijms20174076 (PMC6747156; doi:10.3390/ijms20174076)
Supplement: Supplementary file 1 [file ijms-20-04076-s001.zip › PKP2 Het_Table1(2).pdf]

Table S1

Mouse data

	3 Months		6 Months		Running		Sham		TAC	
	WT	PKP2-Hz	WT	PKP2-Hz	WT	PKP2-Hz	WT	PKP2-Hz	WT	PKP2-Hz
n	8	8	10	10	5	5	5	8	8	9
Male(Female)	1(7)	4(4)	7 (3)	4 (6)	1 (4)	3 (2)	4 (1)	4 (4)	6 (2)	4 (5)
Heart weight (g)	0.135 ± 0.016	0.1651 ± 0.043	0.171 ± 0.041	0.159 ± 0.043	0.15 ± 0.006	0.16 ± 0.0084	0.18 ± 0.02	0.15 ± 0.02	0.25 ± 0.07	0.23 ± 0.05***
Body weight (g)	22.0 ± 2.9	25.7 ± 4.2	32.9 ± 6.8	29.7 ± 6.2	23.7 ± 1,6	25.8 ± 2,1	30.86 ± 2.2	25.51 ± 3.6	28.92 ± 3.5	27.25 ± 4.1
HW/BW (g/g)	6.16 ± 0.60	6.36 ± 0.81	5.17 ± 0.58	5.37 ± 0.91	0.63 ± 0.4	0.62 ± 0.4	5.95 ± 0.26	5.84 ± 0.92	8.60 ± 1.99[#]	8.44 ± 1.85**
Tibea lenght (g)	1.76 ± 0.05	1.81 ± 0,04	1.89 ± 0.07	1.88 ± 0.04	1.83 ± 0.04	1.82 ± 0.08	1.88 ± 0.03	1.81 ± 0.03	1.85 ± 0.04	1.85 ± 0.04*
HW/TL (g/cm)	0.077 ± 0.008	0.091 ± 0.022	0.09 ± 0.02	0.08 ± 0.02	0.082 ± 0.003	0.088 ± 0,005*	0.096 ± 0.008	0.082 ± 0.013	0.135 ± 0.035	0.123 ± 0.027***
