# Supplementary material for: Plakophilin-2 Haploinsufficiency Causes Calcium Handling Deficits and Modulates the Cardiac Response Towards Stress
Source: Int J Mol Sci. 2019 Aug 21;20(17):4076. doi: 10.3390/ijms20174076 (PMC6747156; doi:10.3390/ijms20174076)
Supplement: Supplementary file 1 [file ijms-20-04076-s001.zip › PKP2 Het_Table2(2).pdf]

Table S2

Electrophysiological parameters

	3 Months#		6 Months		Running		Sham		TAC		6 Week EAM#	
	WT	PKP2	WT	PKP2	WT	PKP2	WT	PKP2	WT	PKP2	WT	PKP2
n	8	8	10	10	5	5	5	8	8	9	6	6
RR (ms)	128.3 ± 13.9	135.9 ± 50.4	130.6 ± 17.5	134.9 ± 20.3	128.6 ± 14.7	117.2 ± 18.0	119.1 ± 15.4	135.1 ± 16.0	122.7 ± 18.0	113.2 ± 9.6	116.7 ± 5.22	129.9 ± 18.9
HR (BPM)	472.5 ± 50.5	446.3± 50.2	454.9 ± 75.1	466.0 ± 53,6	471.9 ± 58.0	521.8 ± 82.2	510.6 ± 63.6	450.0 ± 55.7	498.7 ± 76.3	533.3 ± 44.3	514.9 ± 21.9	467.2 ± 52.7
P (ms)	10.7 ± 1.3	10.7 ± 2.9	13.4 ± 1.9	12.2 ± 1.4	13.2 ± 2.4	15.6 ± 2.5	13.1 ± 1.8	12.7 ± 1.3	13.7 ± 3.5	12.8 ± 3.7	14.2 ± 4.7	12.8 ± 3.2
PR (ms)	41.1 ± 2.2	40.5 ± 3.7	40.9 ± 3.2	41.3 ± 3.3	39.7 ± 2.5	42.1 ± 2.2	39.7 ± 4.6	39.3 ± 3.0	41.9 ± 3.5	42.1 ± 2.5	31.16 ± 4.05	29.4 ± 6.67
QRS (ms)	11.4 ± 1.1	12.0 ± 1.5	12.2 ± 1.2	11,8 ± 1,2	11.0 ± 0.9	11.6 ± 1.5	9.2 ± 0.5	8.7 ± 0.4	11.0 ± 0.9*	11.5 ± 0.6*	11.58 ± 1.53	11.2 ± 1.44
QT (ms)	49.4 ± 7.2	52.4 ± 3.6	51.8 ± 3.1	50,4 ± 3,1	55.0 ± 9.8	59.3 ± 4.5	56.7 ± 6.6	56.3 ± 5.2	64.1 ± 13.9	59.2 ± 8.6	23.0 ± 3.32	21.6 ± 2.05
QTc (ms)	42.8 ± 6.2	42.82 ± 2.9	44.8 ± 2.0	44.4 ± 3.9	49.0 ± 10.6	55.3 ± 7.4	52.3 ± 8.0	48.7 ± 4.9	57.7 ± 10.1	55.8 ± 9.01	21.3 ± 3.16	19.0 ± 2.20

Data analyzed individually, only comparison between WT and PKP2 possible
