# Supplementary material for: Plakophilin-2 Haploinsufficiency Causes Calcium Handling Deficits and Modulates the Cardiac Response Towards Stress
Source: Int J Mol Sci. 2019 Aug 21;20(17):4076. doi: 10.3390/ijms20174076 (PMC6747156; doi:10.3390/ijms20174076)
Supplement: Supplementary file 1 [file ijms-20-04076-s001.zip › PKP2 Het_Table3(2).pdf]

Table S3

Protein	Gene	Assay ID
Collagen 1α 1	*Col1α 1*	Mm00801666_91
Collagen 1α 2	*Col1α 2*	Mm00483888_m1
MMP-9	*Mmp9*	MM00442991_m1
TIMP-1	*Timp1*	MM00441818_m1
NF-κβ	*NF-κβ*	MM00477798_m1
IL-6	*Il6*	MM00446190_m1
