# Supplementary figures and images for: Plakophilin-2 Haploinsufficiency Causes Calcium Handling Deficits and Modulates the Cardiac Response Towards Stress
Source: Int J Mol Sci. 2019 Aug 21;20(17):4076. doi: 10.3390/ijms20174076 (PMC6747156; doi:10.3390/ijms20174076)

Figure S1

A

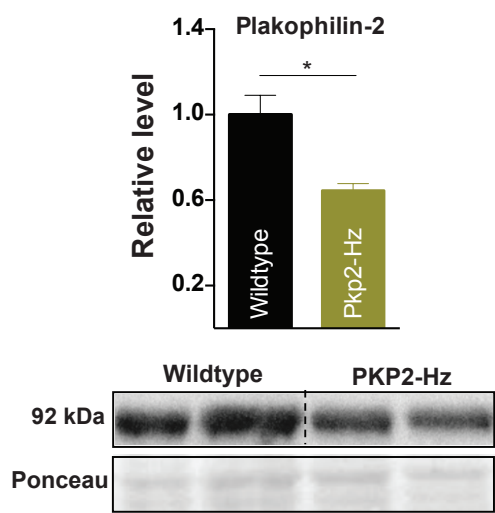

B

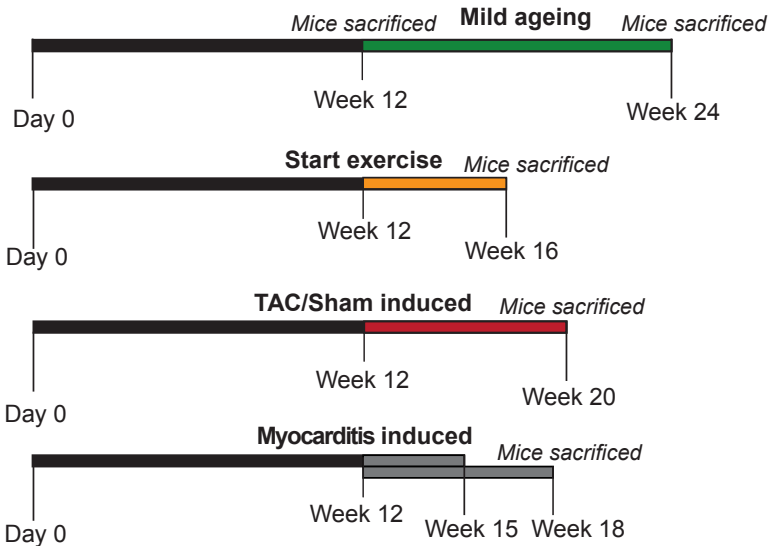

Supplement: Supplementary file 1 [file ijms-20-04076-s001.zip › PKP2 Het_Figure S1.pdf]

Figure S2

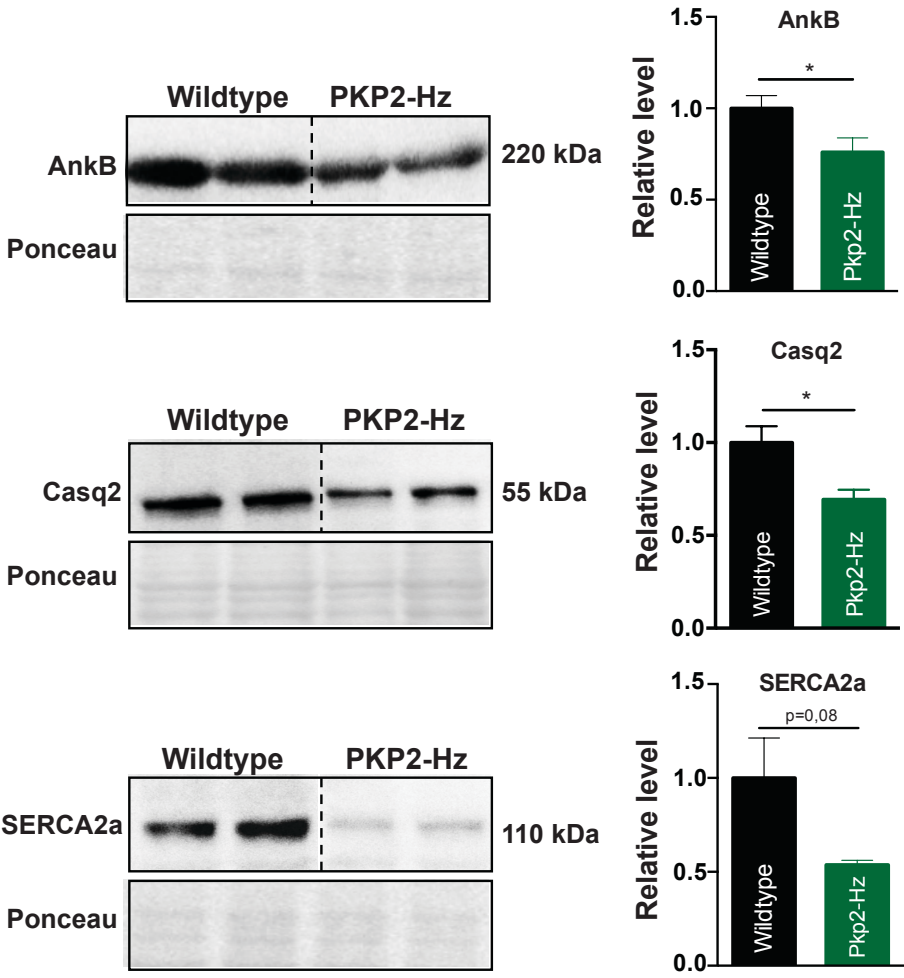

Supplement: Supplementary file 1 [file ijms-20-04076-s001.zip › PKP2 Het_Figure S2.pdf]

Figure S3

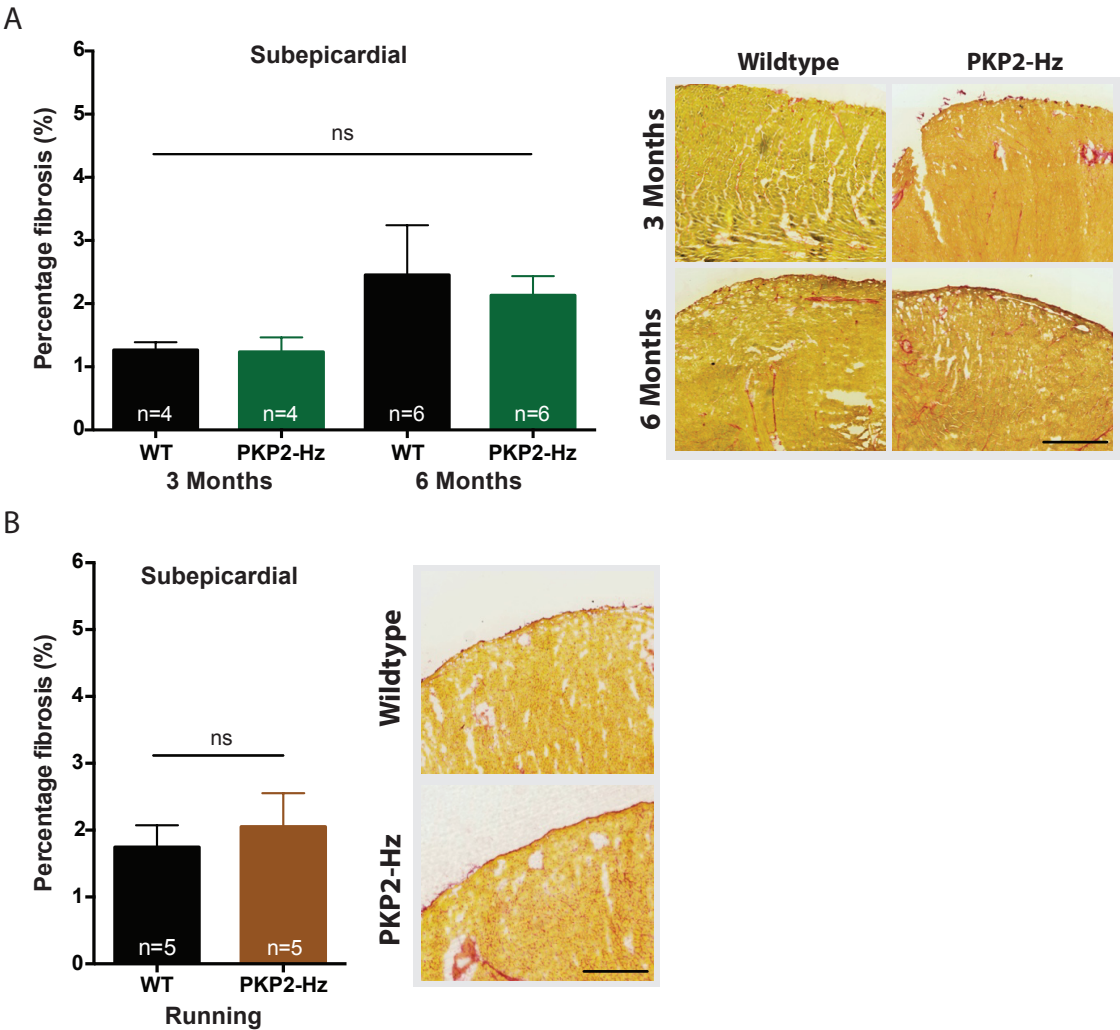

Supplement: Supplementary file 1 [file ijms-20-04076-s001.zip › PKP2 Het_Figure S3.pdf]

Figure S4

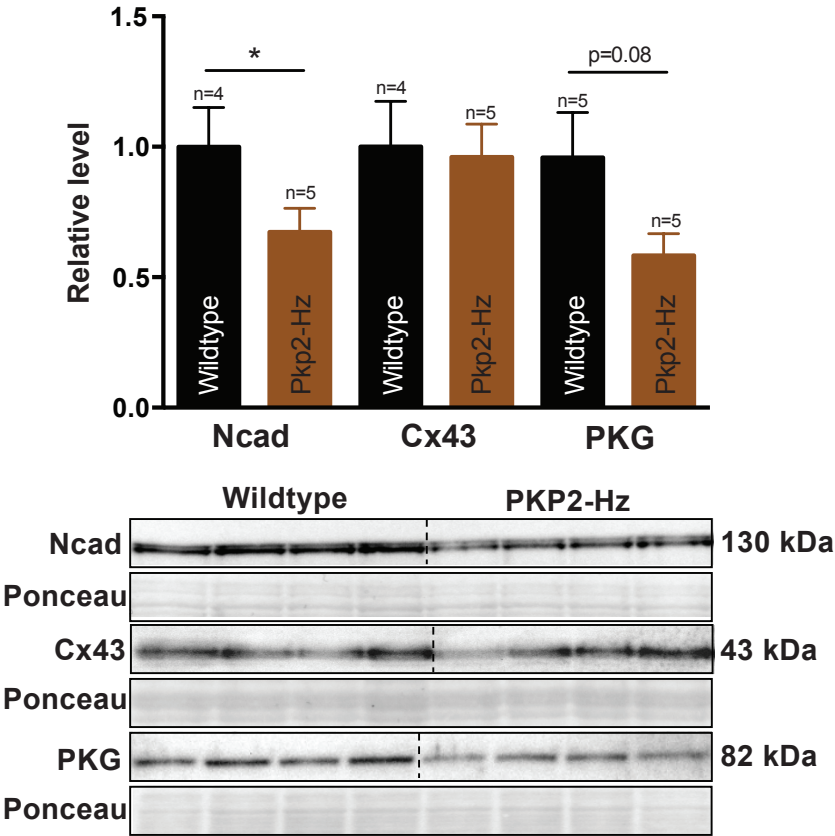

Supplement: Supplementary file 1 [file ijms-20-04076-s001.zip › PKP2 Het_Figure S4.pdf]

Figure S5

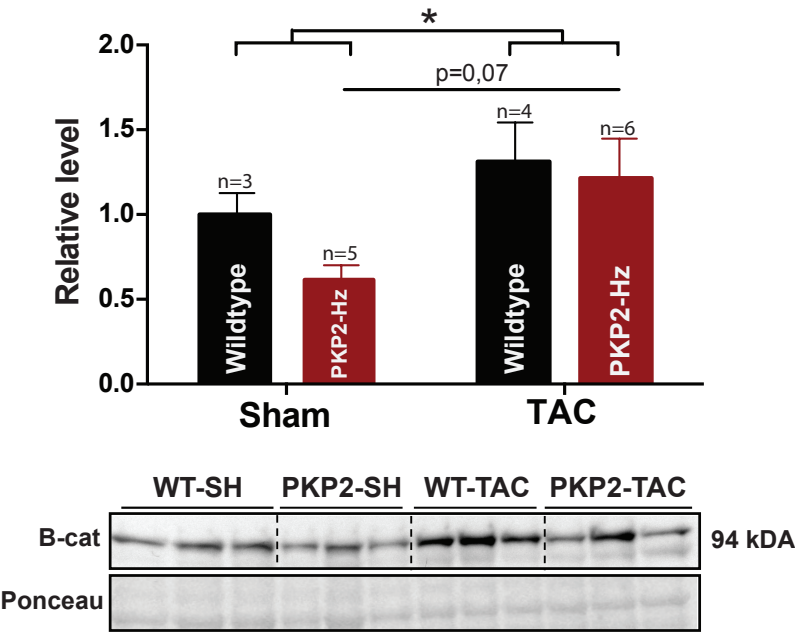

Supplement: Supplementary file 1 [file ijms-20-04076-s001.zip › PKP2 Het_Figure S5.pdf]

Figure S6

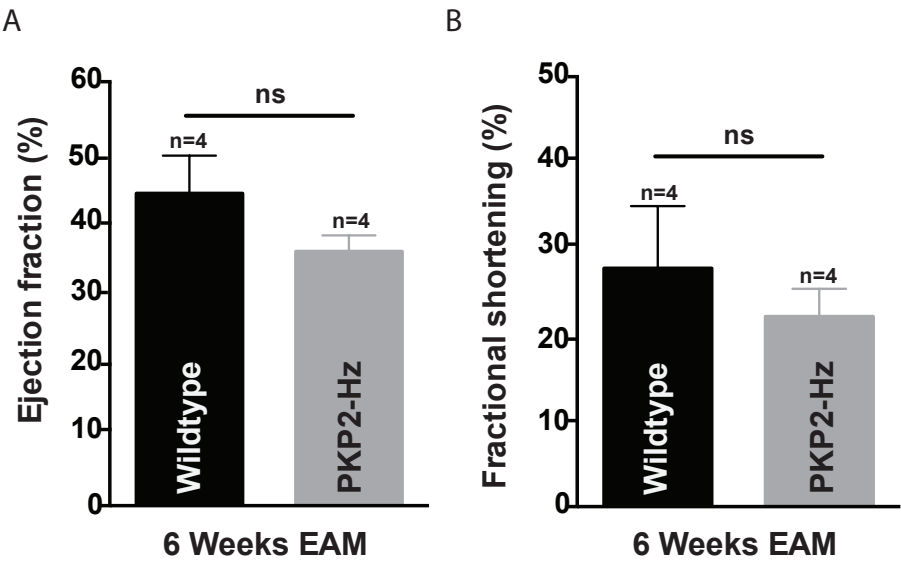

Supplement: Supplementary file 1 [file ijms-20-04076-s001.zip › PKP2 Het_Figure S6.pdf]
